# A Review on the Protective Effects of Honey against Metabolic Syndrome

**DOI:** 10.3390/nu10081009

**Published:** 2018-08-02

**Authors:** Nur Zuliani Ramli, Kok-Yong Chin, Khairul Anwar Zarkasi, Fairus Ahmad

**Affiliations:** 1 Department of Anatomy, Faculty of Medicine, UKM Medical Centre, Universiti Kebangsaan Malaysia, 56000 Cheras, Kuala Lumpur, Malaysia; nurzuliani@ums.edu.my; 2 Department of Biomedical Sciences and Therapeutics, Faculty of Medicine and Health Sciences, Universiti Malaysia Sabah, 88400 Kota Kinabalu, Sabah, Malaysia; khairul.anwar@ums.edu.my; 3 Department of Pharmacology, Faculty of Medicine, UKM Medical Centre, Universiti Kebangsaan Malaysia, 56000 Cheras, Kuala Lumpur, Malaysia; chinkokyong@ppukm.ukm.edu.my; 4 Department of Biochemistry, Faculty of Medicine, UKM Medical Centre, Universiti Kebangsaan Malaysia, 56000 Cheras, Kuala Lumpur, Malaysia

**Keywords:** honey, diabetes mellitus, dyslipidemia, hypertension, obesity

## Abstract

Metabolic syndrome (MetS) is a cluster of diseases comprising of obesity, diabetes mellitus, dyslipidemia, and hypertension. There are numerous pre-clinical as well as human studies reporting the protective effects of honey against MetS. Honey is a nutritional food low in glycemic index. Honey intake reduces blood sugar levels and prevents excessive weight gain. It also improves lipid metabolism by reducing total cholesterol (TC), triglyceride (TG), low-density lipoprotein (LDL) and increasing high-density lipoprotein (HDL), which leads to decreased risk of atherogenesis. In addition, honey enhances insulin sensitivity that further stabilizes blood glucose levels and protects the pancreas from overstimulation brought on by insulin resistance. Furthermore, antioxidative properties of honey help in reducing oxidative stress, which is one of the central mechanisms in MetS. Lastly, honey protects the vasculature from endothelial dysfunction and remodelling. Therefore, there is a strong potential for honey supplementation to be integrated into the management of MetS, both as preventive as well as adjunct therapeutic agents.

## 1. Introduction

Metabolic syndrome (MetS) is a cluster of multiple risk factors that predispose patients to cardiovascular diseases and diabetes [1]. It was first described as syndrome “X” in 1988 by Reaven [2]. Since then, various organizations have established their own diagnostic criteria and definitions including the modified World Health Organization criteria in 1998, the International Diabetes Federation/National Heart, Lung and Blood Institute/American Heart Association criteria (IDF/NHLBI/AHA), the revised National Cholesterol Education Program (NCEP ATPIII) and the Joint Interim Statement or “Harmonized” criteria [1,3,4,5]. According to the latest guidelines from the harmonized criteria published in 2009, MetS is diagnosed when a patient has at least three of the following five conditions which include: (1) Fasting serum glucose ≥100 mg/dL (or receiving drug therapy for hyperglycemia); (2) Blood pressure ≥130/85 mmHg (or receiving medical therapy for hypertension); (3) Serum triglycerides (TG) ≥ 150 mg/dL (or receiving drug therapy for hypertriglyceridemia); (4) Serum high-density lipoprotein cholesterol (HDL-C) < 40 mg/dL in men or <50 mg/dL in women (or receiving drug therapy for reduced HDL-C); and (5) Waist circumference ≥102 cm (40 inch) in men or ≥88 cm (35 inch) in women (for Asian American: ≥90 cm (35 inch) in men or ≥80 cm (32 inch) in women) [1].

Global prevalence of MetS varies according to age, ethnicity and gender. In the United States, the overall prevalence of MetS was 33% within the period of 2003–2012, affecting predominately women and the Hispanic population [6]. The prevalence of MetS among adults in the Asia Pacific region was reported to be approximately 25.7%. Among South-East Asian countries, Malaysia was found to have a higher prevalence of MetS, which stands at 34.3% as compared to Philippines (11.9%), Indonesia (28.4%) and Singapore (20.2%) [7]. The syndrome was generally more prominent among urbanites, females, elderly (≥50 years old) and among the Indian population [8]. Rapid urbanization and changes in lifestyle, especially in developing countries, are closely related to MetS. Some of the risk factors include sedentary lifestyle, consumption of an unhealthy diet consisting of high saturated fat with low fibre, as well as general and central (or abdominal) obesity. Additionally, chronic medical conditions and genetic predisposition to obesity, insulin resistance, diabetes mellitus, high blood pressure and hyperlipidemia also predispose an individual to MetS [9].

Lifestyle modification remains the main emphasis of MetS treatment, with the primary goal to reduce the risk for complications, like atherosclerotic disease [5]. If unsuccessful, the patient may be treated with drug therapy targeting each component of MetS, resulting in polypharmacy [9]. Polypharmacy poses certain challenges to the patients, including non-compliance to medications, adverse drug reactions, drug-to-drug interactions and the requirement for multiple visits to the physician [10]. Hence, prevention is the most feasible option to control and decrease the prevalence of MetS.

Natural products such as ω-3 polyunsaturated fatty acids found in fish, flaxseed oil, resveratrol in red wine, dark chocolate, cinnamon extract, soy protein, dietary fibre, chromium and traditional Chinese herbs have been used as dietary interventions in lowering the incidence of MetS [11]. Generally, these natural products improved MetS through their anti-inflammatory, anti-oxidant, hypoglycemic and hypolipidemic effects. In agreement with this, several studies have documented that honey exerts similar beneficial effects that include anti-oxidant, anti-inflammatory, pancreatoprotective, anti-hypertensive, hepatoprotective and anti-obesity properties [12,13,14,15,16].

According to the Codex Alimentarius, “honey is the natural sweet substance produced by *Apis mellifera* bees from the nectar of plants, secretions of living parts of plants, or excretions of plant-sucking insects on the living parts of plants, which the bees collect, transform by combining with specific substances of their own, deposit, dehydrate, store and leave in the honeycomb to ripen and mature” [17]. However, other species of bees and insects can also produce honey such as stingless bees (*Melipona* and *Trigona* spp.) and Nectarina wasps in South America, as well as several species of honey ants, especially *Melophorus inflatus* in Australia. In addition, social wasps and bumblebees (*Bombus* spp.) also produce small amounts of honey [18]. 

Honey is divided into two broad categories that include Blossom Honey or Nectar Honey (originates from nectars of plants) and Honeydew Honey [mainly from excretions of plant sucking insects (Hemiptera) on the living parts of plants or secretions of living parts of plants] [17]. Additionally, honeydew honey can also be defined as a type of honey produced by sap-sucking insects (e.g., aphids) and is made from honeydew, but not from the blossom nectar [19]. Honey can be further classified as mono- or multifloral honey according to the quantification of pollen types. There are four types of pollen quantification: (1) Predominant pollen types (calculated total pollen grains >45%); (2) Secondary pollen types (16–45%); (3) Important minor pollen types (3–15%); and (4) Minor pollen types (<3%). The honey is considered as monofloral if it has a predominant pollen type. In contrast, if the honey contains other pollen types, it is classified as multifloral [20,21]. 

The composition, flavour and aroma of the honey depend on the plant sources, climate and environmental conditions. Carbohydrate is the main constituent in dry weight honey with fructose being the highest component at approximately 32–38% followed by glucose as well as other disaccharides and oligosaccharides. Honey also contains organic acids, minerals, trace elements, numerous vitamins, enzymes and proteins [22,23,24,25]. The antioxidant capability of honey is linked to its polyphenol compounds which comprise of flavonoids (e.g., quercetin, luteolin, kaempferol, apigenin, chrysin, galangin), phenolic acids, antioxidant enzymes (e.g., glucose oxidase and catalase), ascorbic acid, and carotenoid [23,26,27]. 

Different types of honey exert different degrees of health benefits even though they have common composition and physicochemical properties such as high osmolarity, low moisture and acidity. This is related to its geographical, seasonal and botanical origin as well as the harvesting, processing and storage conditions [28,29]. This review aimed at illustrating the pathogenesis of MetS and to explore the beneficial effects of honey on each component of the disease. 

## 2. Anti-Obesity Effects

Obesity is the central occurrence in MetS. According to the harmonized criteria mentioned previously, the presence of three out of five criteria in an individual is required for diagnosing MetS. One of the criteria is central obesity due to the accumulation of visceral adipose, defined by increased waist circumference [1]. As excessive, unused energy is being stored in the body, adipose tissue becomes hypertrophied and undergoes hyperplasia, leading to reduced blood supply to the tissue, resulting in a hypoxic environment. At the same time, the adipose tissue itself produces a number of pro-inflammatory mediators namely tumour necrosis factor-α (TNF-α), interleukin-6 (IL-6), leptin, resistin and plasminogen activator inhibitor-1 (PAI-1) [9]. These inflammatory factors promote inflammation and macrophage infiltration leading to the manifestation of other MetS components.

Protective effects of honey against obesity have been reported in animal experimental studies (Table 1). For instance, short-term feeding with honeydew honey (*Northofagus solandrii*) resulted in a lower percentage of weight gain in rats than in those fed with sucrose and mixed sugars diet, although there were no differences in term of total energy intake among the rats [30]. Likewise, long-term administration of honeydew honey for 365 days significantly prevented overall weight gain in adult rats compared to the rats receiving long-term sucrose. In addition, rats receiving long-term honey treatment developed significantly lower body fat percentage when analysed with dual-energy X-ray absorptiometry (DEXA) scan compared to the sucrose group, measuring at 25.5 ± 6.1% and 34.7 ± 9.1% of body fat percentage, respectively [31]. Like adult rats, a similar result has been observed in growing rats. By feeding 7-day old rats with either cane syrup or honey with identical nutritional value, visceral fat of rats receiving cane syrup was substantially higher as compared to rats fed with honey. In addition, the liver of cane syrup-fed rats showed accumulation of fat droplets as well as fatty degeneration which were significantly reduced in the honey supplemented group [32]. The anti-obesity effect of honey was further demonstrated by Nemoseck et al. (2011). In this experiment, the researchers reported that rats given a diet containing 20% carbohydrate from clover honey developed markedly lower weight gain as well as significantly reduced fat pad weight than rats given an isoenergetic diet from liquid sucrose [16].

It is noteworthy that honey is also capable of exerting anti-obesity effects in human as reported in randomized clinical trials (RCTs). A total number of 55 apparently healthy, overweight and obese individuals with body mass index (BMI) of >25 kg/m^2^ were recruited and allocated into either sucrose-receiving or honey-receiving groups randomly. At the end of study duration, individuals prescribed with 70 g honey had a mild reduction in body weight (1.3%), fat weight (1.1%) and body fat percentage (1.8%) along with significantly decreased BMI (1.2%; *p* = 0.02) [33]. In another RCT, Bahrami et al. (2009) recruited 42 patients with type II diabetes mellitus and randomly assigned them into control and honey-supplemented groups for 56 days. This study reported that the addition of honey in the diabetic treatment regime significantly reduced the patients’ body weight (*p* = 0.000) at the end of the study period [34].

## 3. Antidiabetic Effects

MetS is predictive of type II diabetes [35]. Individuals with metabolic syndrome had a five-fold increased risk for developing the disease [1]. Type II diabetes mellitus is a chronic noncommunicable disease characterized by insulin insensitivity causing a rise in the blood glucose level. It affects nearly 150 million of the world population and the number is expected to double by the year 2025 [36]. People with diabetes have increased risk of coronary heart disease and stroke, contributing up to 10% of adult mortality in developed nations [37]. Current management of type II diabetes in clinical settings include mass screening of the general population, prevention via lifestyle modification and weight reduction; with pharmacological treatment as the last resort if other steps have failed [38]. For years, researchers have been conducting various animal studies, preclinical trials as well as RCTs to show the beneficial effects of honey on diabetes (Table 2). 

Streptozotocin (STZ) is known to induce diabetes in rats by damaging pancreatic β-cells of islets of Langerhans [39]. These cells are responsible for the production of insulin to increase cellular uptake of glucose. Administration of mad honey (produced from the flower of *Rhododendron* spp., a botanical family of Ericaceae) in STZ-induced diabetic rats and non-diabetic rats for 3 days has been shown to markedly reduce blood glucose levels [40,41]. Antidiabetic effects of honey are also explained by its ability to modulate adiponectin levels as well as its antioxidant capacity. Adiponectin, a hormone secreted by adipose tissue to regulate glucose and lipid metabolism, is found to be decreased in diabetic patients [42]. Meanwhile, oxidative stress-mediated lipid peroxidation has been linked to the development of complications in diabetes [43,44]. A high level of adiponectin reduces systemic inflammation and improve insulin sensitivity [45]. In a study by Hemmati et al. (2015), STZ-induced diabetic rats were orally fed with honey for 21 days at the doses of 1.0 and 2.0 g/kg/day. The researchers observed a significant increase of adiponectin levels (4.5 ± 0.2 and 4.2 ± 0.3 mg/L, respectively) with a marked decrease of malondialdehyde (MDA) levels in the supplemented rats compared to the diabetic control rats. Thus, these effects were correlated with a significant improvement of fasting blood sugar (FBS) levels and lipid profiles in honey-supplemented diabetic rats [46]. 

Hypoglycemic effect of stingless bee honey (SLBH) *G. thoracica* has been demonstrated by Aziz et al. (2017) in partial insulin deficiency rats induced by combined STZ-nicotinamide administration. Rats treated with SLBH at the dose of 1.0 and 2.0 g/kg/day for 28 days showed a significant reduction of FBS level compared to the untreated diabetic rats, attributed to a notable improvement in serum insulin level. Concurrently, the treatment significantly increased anti-oxidative enzyme catalase (CAT) expression in the immunohistochemical analysis, which reduced oxidative stress in the pancreas and promoted pancreatic healing process [13]. An analysis of SLBH via liquid chromatography-mass spectrometry (LC-MS), showed that the presence of l-phenylalanine in honey was found to be responsible for stimulating insulin release and improved glucose tolerance in diabetic rats [13,47].

Tualang honey (TH) is a type of Malaysian multifloral jungle honey produced by *Apis dorsata* (or rock bees) that lives in hives built on high branches of *Kompassia excelsa* (known locally as Tualang tree) that are mainly found in tropical rain forest [48]. TH has been studied for its anti-oxidant effect in the pancreas of STZ-induced diabetic rats. TH (1.0 g/kg/day) given for 28 days to diabetic rats resulted in significant downregulation of pancreatic superoxide dismutase (SOD) and MDA (*p* < 0.01) along with elevated pancreatic CAT activity (*p* < 0.05) compared to diabetic control rats. Oxidative damage in the pancreas of diabetic rats was protected by the anti-oxidant effects of TH, leading to significant improvement of FBS in diabetic rats compared to diabetic control (median (IQR): 8.8 (5.8) and 17.9 (2.6) mmol/L respectively) [49]. Other types of honey, such as Nigerian honey, also exert a similar hypoglycemic effect. When given to alloxan-induced diabetic rats for 21 days with a dose of 1.0 and 2.0 g/kg/day, diabetic rats fed with honey had significantly reduced FBS compared with the diabetic control (*p* < 0.05) [50]. Shorter duration of honey supplementation for a period of 7 days to alloxan-induced diabetic rats also reported a similar trend although the results were not statistically significant (alloxan + honey vs. alloxan alone; FBS mean ± SD; 8.44 ± 1.66 vs. 11.05 ± 2.11 mmol/L, respectively; 2-h postprandial glucose level: 11.57 ± 2.22 vs. 16.45 ± 3.11 mmol/L, respectively) [51].

Some types of honey required a longer duration of treatment to achieve a significant antidiabetic effect. A study by Arabmoazzen et al. (2015) administered 3 mL/kg of 5% honey solution three times per day for 56 days consecutively to noise-induced diabetic rats. At the end of the study period, serum glucose concentration in the diabetic rats treated with honey was significantly lower compared to the untreated diabetic rats (208 ± 34.6 and 401 ± 25.9 mg/dL, respectively; *p* < 0.01). In addition, histological analysis showed that rats treated with honey had a significantly higher number of pancreatic β-cells [52]. This effect was supported by a human study as well. Unprocessed honey was given orally to 25 type II diabetic patients at the starting dose of 1.0 g/kg/day. The dose was gradually increased by 0.5 g/kg/day every 14 days for a total duration of 56 days. Patients receiving honey were observed to experience a significant reduction of FBS level at the end of treatment compared to the baseline level (124.3 ± 37.5 vs. 153.3 ± 43.9 mg/dL, respectively; *p* = 0.01) [34]. An instantaneous effect of honey was also reported when seven type II diabetic patients were given honey solution (90 g of honey dissolved in 250 mL of water) as a substitute for dextrose 30 min prior to blood sampling for oral glucose tolerance test. The patients had significantly lowered blood glucose elevation when administered with honey compared to dextrose [53]. This showed that substituting honey for sugar may be useful in the management of diabetes if taken in moderate quantities as honey does not only reduce blood glucose, it also ameliorates the risk factors for cardiovascular and metabolic diseases [12,54,55]. 

The mechanism by which honey lowers blood glucose lies on its ability to inhibit α-amylase and α-glucosidase activities. Alpha-amylase is an enzyme responsible for hydrolysis of complex starch to oligosaccharides, whereas α-glucosidase hydrolyses oligosaccharides, trisaccharides and disaccharides into monosaccharides as the end product. It was well documented that inhibition of these enzymes was able to lower postprandial blood glucose levels [56,57,58]. In this regard, antidiabetic activity of honey was analyzed using in vitro α-amylase and α-glucosidase enzyme inhibition assays by Krishnasree & Mary (2017). *Trigona iridipennis* honey, a type of SLBH honey, had the strongest α-amylase and α-glucosidase inhibitory properties compared to other multifloral honey species. This was comparable to standard diabetic therapy by acarbose especially at the highest concentration of 500 μg/mL. Furthermore, raw *T. iridipennis* honey had the lowest glycemic index (GI) of 55, making it a suitable option as a sweetener for diabetic patients [59].

## 4. Hypolipidemic Effects

Atherogenic dyslipidemia is one of the core metabolic risk factors of MetS. It consists of raised serum TG and apolipoprotein B (apo B)-containing lipoproteins that include low-density lipoprotein (LDL) and very low-density lipoprotein (VLDL) as well as reduced levels of serum HDL [9,60,61]. Increased upper body fat (whether visceral or subcutaneous) contributes to the amount of circulating free fatty acids (FFA) in the systemic circulation and portal circulation to the liver. The majority of portal FFA originates from the systemic FFA [62,63]. Excess visceral fat in obese persons, termed as ectopic fat, will be deposited in the liver, heart and skeletal muscle, which results in insulin resistance and disinhibition of lipolysis [11,60,64,65]. As a consequence, there will be an increased flow of FFA from visceral adipose tissue to the liver through splanchnic circulation [60,61]. Eventually, excess FFA in the liver will be used to produce TG that will be incorporated into VLDL. The TG in VLDL is then transferred to HDL in exchange for cholesteryl esters. This TG-enriched HDL is then rapidly cleared by the hepatic lipase, which leads to a reduced amount of HDL in the circulation [60,61,64,66]. At the same time, cholesterol-rich VLDL will be converted to LDL and gets deposited in the intimal layer of the vascular wall. Due to increased oxidative stress in obese individuals, the deposited LDL is oxidized and triggers inflammatory responses resulting in atherogenesis [67]. In the end, this pathophysiology increases the risk of atherosclerotic cardiovascular diseases (ASCVD) [68].

Honey has the capacity to ameliorate cardiovascular risk factors as shown by Yaghoobi et al. (2008) (Table 3). In this experiment, obese subjects were given 70 g of honey dissolved in 250 mL tap water daily for 30 days vs. sucrose in the control group. Honey was able to markedly lower the TG levels by 19% (*p* = 0.006) and non-significantly reduced TC by 3.3% and LDL by 4.3% in dyslipidemic subjects. In contrast, honey non-significantly reduced TC (3%), LDL (5.8%), TG (11%), and increased HDL (3.3%) in non-dyslipidemic individuals [33]. Furthermore, this effect was supported by an RCT done by Bahrami et al. (2009). The study reported that after ingesting honey for 56 days, there was a notable decrease in TC, LDL and TG (*p* = 0.000) with increased HDL concentration compared to the baseline level (*p* < 0.05) in diabetic patients [34]. Another RCT concluded that supplementation of honey for 28 days significantly reduced TC and LDL together with increased HDL. However, these effects were strongly correlated with gender, BMI and ethnicity [69].

Hypolipidemic effect of honey was further demonstrated by Al-Waili (2004). There was a significant reduction of TC (8%) as well as a non-significant decrease of LDL (11%) among honey supplemented dyslipidemic patients [53]. In another study using a larger sample size of 60 hypercholesterolemic patients, supplementation with 75 g mixed blossom honey solution for 14 days was compared with sugar solution. This study discovered that honey significantly reduced LDL cholesterol, especially in female patients, although the effect of honey on other lipid profile parameters was less pronounced [70]. 

Findings from the animal experimental model of hyperlipidemia were found to complement the hypolipidemic properties of honey in human subjects. By using STZ-induced diabetic rats, Öztaşan et al. (2005) documented that administration of mad honey at the dose of 50 mg/kg for 3 days caused significant reductions of TC, TG and VLDL. Since the effects were so pronounced, they suggested that the underlying mechanism was due to the action of mad honey on the parasympathetic nervous system (or M_2_-muscarinic receptors) that increases insulin release from pancreatic Langerhans cells. This indirectly resulted in a reduction of lipid profiles [40]. Another animal study reported that Nigerian honey administration for 21 days to rats with alloxan-induced diabetes mellitus produced a significant reduction of TG, non-HDL cholesterol especially VLDL, cardiovascular risk index (CVRI) (CVRI = TG/HDL) and coronary risk index (CRI) (CRI = TC/HDL) (*p* < 0.05). On the other hand, it markedly elevated HDL cholesterol along with nonsignificant reduction of LDL, TC and atherogenic index (AI) (AI = LDL/HDL), especially at the dose of 1.0 & 2.0 g/kg compared with the non-diabetic controls [50]. 

A diet containing high fructose induces dyslipidemia and exerts pro-oxidant effects [71,72]. Replacement of honey with refined carbohydrate in the purified diets of rats for 14 days has shown to significantly lower TG levels (1.49 ± 0.12 mmol/L) in comparison to fructose-fed rats (2.03 ± 0.20 mmol/L) (*p* < 0.05). At the same time, the heart homogenates showed significantly higher thiobarbituric acid-reactive substances (TBARS) (+58%) in rats fed with fructose compared with the honey and control groups [55]. This was also supported by another study by Nemoseck et al. (2011) whereby clover honey was substituted with sucrose in the diet containing a similar amount of energy density for 33 days. Honey-fed rats had 29.6% lower TG levels than the sucrose-fed rats, (54.4 ± 19.3 mg/dL vs. 77.2 ± 36.7 mg/dL respectively) (*p* ≤ 0.05) [16]. Comparably, a study with a longer treatment period was conducted, whereby treatment of 10% honeydew honey (100 g/kg) mixed in diets was compared with sucrose for 365 days. Although there was no significant decrease in TG and LDL, the honey-fed rats had significantly higher HDL levels (2.82 ± 0.30 mmol/L) than the rats on a sugar-free diet (2.32 ± 0.33 mmol/L) and sucrose diet (2.44 ± 0.51 mmol/L) [31]. 

Apo-B and triglyceride-rich lipoproteins play a role in the development of ASCVD [68,73]. Pretreatment with 3 g/kg/day of TH for 45 days in rats with isoproterenol (ISO)-induced myocardial infarction was shown to normalize cholesterol levels. Serum TC and TG levels were significantly reduced in rats receiving TH compared to ISO alone (*p* < 0.05) [74]. Studies by Aziz et al. (2017) observed similar results, in which STZ-nicotinamide-induced rats given SLBH for 28 days showed marked reductions of TC, TG and LDL levels along with a notable increase in HDL level compared to untreated diabetic rats at the dose of 2.0 g/kg (*p* < 0.05) [13].

## 5. Antihypertensive Effects

Hypertension is one of the components of MetS. There are many factors that contribute to the development of hypertension and one of them is obesity-related hypertension [75]. Blood pressure is regulated by the autonomic nervous system by means of controlling the diameter of blood vessels via vasodilation and vasoconstrictions [76]. Centrally obese individuals have insulin resistance with a compensatory increase in insulin secretion, leading to hyperinsulinemia. As a consequence, there will be activation of the sympathetic nervous system (SNS), through stimulation of α- and β-receptors. Vasoconstriction of blood vessels, increased cardiac output by the heart as well as sodium retention by the renal tubules are major effects of SNS activation in relation to hypertension [77]. Leptin, an appetite-suppressing hormone secreted by adipocytes, is increased in obese individuals [78,79]. Leptin has also been reported to stimulate SNS, thus contributing to elevated blood pressure associated with obesity [80]. Another mechanism by which leptin increases blood pressure is through the regulation of neuronal circuits in the dorsomedial hypothalamus (DMH). A study conducted in diet-induced obesity in mice found that injection with leptin receptor antagonist at the DMH for 7 days resulted in a significant reduction of heart rate and systolic blood pressure [79]. It is also evident that the renin-angiotensin system constituents such as angiotensinogen (AGT), renin, angiotensin converting enzyme and angiotensin II receptors were detected in human adipose tissues, as well as in rodents, acting as an endocrine organ [81,82]. 

Another pathological mechanism that contributes to the development of hypertension in MetS includes the renal oxidative stress. In chronic medical conditions like MetS, long-term exposure to reactive oxygen species (ROS), especially to hydrogen peroxide (H_2_O_2_), caused down-regulation of the Akt signalling pathway and impaired nuclear translocation of Nrf2, thereby inhibiting the antioxidant response [83]. TH supplementation in spontaneously hypertensive rats (SHR) at 1 g/kg/day for 84 days was effective in attenuating renal oxidative stress. It upregulated mRNA and protein expression of transcription factor Nrf2 (a master regulator of antioxidation system) and enhanced its nuclear translocation, resulting in induction of antioxidation enzymes such as catalase and glutathione-S-transferase at the level of gene expression. Increased synthesis and activities of these enzymes blunted the effect of ROS in the kidney, which ultimately reduced the blood pressure [14]. The antioxidative properties of honey is supported by another similar study whereby TH supplementation in STZ-induced diabetic SHR for 21 days reduced systolic blood pressure significantly (*p* < 0.01) compared to the untreated diabetic SHR [84].

Oxidative stress causes several other pathological changes, including inflammation of the vascular wall, reduced vasodilatory agent nitric oxide (NO) bioavailability, extracellular matrix alterations as well as increased vascular cell proliferation. Combination of these effects leads to endothelial dysfunction that contributes to the development of hypertension [85]. Association between endothelial dysfunction and hypertension has been documented in a previous study by Dell’Omo et al. (2004). Comparing between normotensive individuals with hypertensive patients as well as hypertensive patients with MetS, they reported that hypertensive patients with MetS developed a significantly higher level of transcapillary escape rate of albumin (TERalb) at 10.9%/h vs. 8%/h in the other two groups (*p* < 0.004) [86]. Furthermore, in an in vitro study, treatment of human umbilical vein endothelial cells with TH prevented the increase of H_2_O_2_-induced endothelial permeability by decreasing the actin remodeling process along with reduced caveolin-1 expression, which was comparable to the effect of Trolox, a water-soluble vitamin E analogue [87]. Apart from that, Al-Waili (2003) reported that there was a high concentration of vasodilatory agent NO in honey that contributes to the therapeutic effects towards hypertension [88].

Diets high in fructose, sucrose and fat have been observed to increase blood pressure [89,90]. As such, a study by Romero-Silva et al. (2011) demonstrated that rats receiving a hypercaloric diet with the addition of 20% honey caused no increase in blood pressure as compared to untreated rats (*p* < 0.05) [91]. The immediate blood pressure-lowering effect of honey has been observed in hypertensive subjects. Administration of 60% honey solution via inhalational route showed a marked decrease in blood pressure at 60 and 120 min post-treatment [92]. The instant hypotensive effects of honey have also been reported by Aluko et al. (2013; 2014) in healthy subjects [93,94]. These studies related to the antihypertensive effects of honey have been summarized in Table 4.

## 6. Fructose vs. Honey

Reducing sugars such as fructose and glucose are present in honey with concentration ranging from 15.8–48.4 g/100 g honey and 9.2–41.9 g/100 g honey, respectively [95]. While there is increasing evidence that honey potentially reverses MetS, it may raise questions whether the beneficial effects of honey might be related to its fructose content.

Based on current knowledge, fructose has a pro-oxidative property that is closely related to the purine metabolism pathway. As fructose enters the cell, it gets phosphorylated and then converted to ribose-5-phosphate (R-5-P) through the pentose phosphate pathway [96,97]. In the purine metabolism, R-5-P serves as the precursor molecule for nucleotides especially adenosine monophosphate (AMP) and guanosine monophosphate (GMP) [98]. The relationship between fructose and the purine metabolism pathway was well documented in the literature. Rapid infusion of 125–200 g of fructose over 3–4 h in human subjects was found to increase uric acid (a metabolite of purine metabolism) turnover by four times [99]. In addition, recent systematic reviews and meta-analyses reported that there was close association between fructose consumption and development of future hyperuricemia that may be explained by increased purine nucleotides production as well as their catabolism [100]. In both studies, there was involvement of xanthine oxidase (XO), an enzyme responsible for oxidation of hypoxanthine—another metabolite of AMP and GMP catabolism—to xanthine followed by conversion of xanthine to uric acid. The biochemical reactions catalyzed by XO release hydrogen peroxide (H_2_O_2_), a reactive oxygen species (ROS), that may further contribute to the high oxidative stress environment in MetS [101].

Indeed, a study on hamster islet tumour (HIT) cells derived from hamster pancreatic β-cells reported that exposure to fructose increased oxidative stress in HIT cells in a time- and dose-dependent manner. Fructose exerts pro-oxidative effects by increasing intracellular H_2_O_2_ concentration, inactivating anti-oxidant protein glutathione peroxidase (GPx) as well as inhibiting GPx mRNA expression [102]. In relation to this, rats fed with 10% fructose drinking water developed increased oxidative stress (denoted by higher concentration of total ROS, H_2_O_2_, superoxide level and lipid peroxidation, activation of XO, as well as decreased activity of SOD and total antioxidant capacity) as compared to the control group. These fructose-induced changes were successfully reversed by allopurinol treatment (an XO inhibitor) at 5 mg/kg/day. Apart from that, fructose-fed rats also had significantly higher body weight, increased hepatic TG and TC concentration, evidence of hepatic inflammation on histological analysis, and hepatic fat accumulation compared to control rats which were also reduced by allopurinol administration [103]. This corroborated with a study which showed male rats fed with 20-25% fructose for 8 weeks exhibited all conditions of MetS and hypertrophy of adipocytes [104]. 

Additionally, fructose consumption induces MetS syndrome through the activation of several other biochemical processes. By utilizing electrospray ionization-tandem mass spectrometry (ESI-MS/MS)-based proteomics, analysis on a MetS rat model revealed that a 60% high-fructose diet triggered more glucose production via gluconeogenesis, promoted accumulation of lipid through activation of enzymes related to fatty acid synthesis, aggravated endoplasmic reticular stress, and increased oxidative stress that resulted in elevated antioxidative mechanism by glutathione *S*-transferase and peroxiredoxin I enzymes, along with the stimulation of inflammatory processes. These changes resulted in hyperglycemia, hypercholesterolemia and hypertension in the fructose-fed rats compared to control [105].

Conversely, fructose concentration in honey is lower, which is less than 50% as mentioned previously. Hence, it will not cause any detrimental effect to health. At the same time, honey has higher contents of enzymatic (e.g., catalase and glucose oxidase) and non-enzymatic anti-oxidants (e.g., ascorbic acid, carotenoids and polyphenols) that are responsible for its anti-oxidative properties [106]. A previous study has also reported that when replacing honey with refined carbohydrates that contain same amount of fructose, the pro-oxidative effect of fructose was significantly attenuated [55]. These evidences provide explanation on why the beneficial effects of honey against MetS are unlikely caused by its fructose contents.

## 7. Conclusions

Honey protects against MetS by exerting anti-obesity, antidiabetic, hypolipidemic and hypotensive activities. The mechanisms underlying these effects include its low GI nature, which limits weight gain and accumulation of fat storage; improvement of insulin sensitivities and lowering of blood glucose levels; enhanced lipid metabolism, leading to prevention of atherogenesis; attenuation of oxidative stress; as well as protection from endothelial dysfunction among many others. Therefore, honey has a strong potential to be utilized in the management of MetS as a preventive and adjunct therapeutic agent.

## Figures and Tables

**Table 1:** Anti-obesity effects of honey in clinical and pre-clinical studies.

Author	Honey Sources/Types	Dose	Duration	Models	Results
Nemoseck et al. (2011) [16]	Monofloral Clover honey	20% honey (300 g/kg) mixed in the diet	33 days	Male Sprague-Dawley rats	Compared to the sucrose-fed group, honey-fed rats were found to have significantly: Lower weight gain (118 ± 20 g vs. 135 ± 33 g). Lesser food intake (729 ± 150 g vs. 880 ± 172 g). Decreased fat pad weight (3.89 ± 0.63 g vs. 4.87 ± 0.94 g). Smaller percentage of relative fat (1.11 ± 0.13% vs. 1.33 ± 0.16%). Reduced level of serum leptin (449.0 ± 143.3 pg/mL vs. 572.4 ± 211.8 pg/mL).
Chepulis (2007) [30]	Honeydew honey (*Northofagus solandrii*)	10% honey (100 g/kg) mixed in the diet	42 days	Sprague-Dawley rats	Compared with sucrose and mixed sugar-fed rat groups, rats receiving honey had a significantly lesser percentage of weight gain (155.0 ± 6.5% and 151.5 ± 14.4% vs. 138.3 ± 11.2%, respectively; *p* = 0.009).
Chepulis & Starkey (2008) [31]	Honeydew honey	10% honey (100 g/kg) mixed in the diet	365 days	Sprague-Dawley rats	Long-term feeding with honey resulted in: Markedly lower percentage of overall weight gain than sucrose-fed rats (107.2 ± 13.8% vs. 130.6 ± 26.7%). Significantly smaller total body fat percentage compared to sucrose-fed rats (25.5 ± 6.4% vs. 34.7 ± 9.1%).
Ajibola et al. (2013) [32]	Monofloral sunflower honey	10% (low dose) or 50% (high dose) honey solution	91 days	Sprague-Dawley rats	Both low and high dose honey administration caused: Significantly lower visceral fat mass and visceral fat percentage (especially in male rats). Protected the liver against the accumulation of fat droplets as well as fatty degeneration in both male and female rats.
Yaghoobi et al. (2008) [33]	Natural unprocessed honey (unknown type)	70 g honey dissolved in 250 mL tap water daily	30 days	60 overweight or obese (BMI > 25 kg/m^2^) individuals	Honey supplementation led to: Non-significant weight loss (1.3%; *p* = 0.09). Mild decreased in body fat percentage (1.1%; *p* = 0.279). Non-significant decreased in fat mass (1.1%; *p* = 0.682). Significant reduction of BMI (1.2%; *p* = 0.02).
Bahrami et al. (2009) [34]	Multifloral natural, unprocessed honey	1st 14 days: 1.0 g/kg/day 2nd 14 days: 1.5 g/kg/day 3rd 14 days: 2.0 g/kg/day 4th 14 days: 2.5 g/kg/day	56 days	48 patients with type II DM (25 in honey group, 23 in control group)	Consumption of honey significantly decreased body weight in type II DM patients as compared to control (69.5 ± 11.9 kg vs. 70.3 ± 8.0 kg; *p* = 0.000).

BMI, body mass index; DM, diabetes mellitus.

**Table 2:** Anti-diabetic effects of honey in clinical and pre-clinical studies.

Author	Honey Sources/Types	Dose	Duration	Models	Results
Aziz et al. (2017) [13]	Stingless bee honey	1.0 or 2.0 g/kg/day	28 days	Male Sprague-Dawley rats	Treatment of 1.0 g/kg/day honey resulted in a modest decrease of fasting blood sugar (FBS) level at the end of study duration. Honey administration at the dose of 2.0 g/kg/day caused marked reduction of FBS after 28 days. Treatment with both 1.0 and 2.0 g/kg/day of honey: Protected against shrinkage of the pancreatic islets sizes. Significantly elevated the serum insulin level. Markedly increased insulin intensity in the pancreatic islets (as seen in the immunohistochemistry analysis).
Bahrami et al. (2009) [34]	Natural, unprocessed honey (obtained from Samans kandeh, Neka, Sari City, Iran)	1st 14 days: 1.0 g/kg/day 2nd 14 days: 1.5 g/kg/day 3rd 14 days: 2.0 g/kg/day 4th 14 days: 2.5 g/kg/day	56 days	48 type II diabetic patients	Patients receiving honey were observed to have significant reduction of fasting blood sugar level after 56 days treatment compared to baseline (124.3 ± 37.5 vs. 153.3 ± 43.9 mg/dL, respectively; *p* = 0.01).
Öztaşan et al. (2005) [40]	Mad honey	50 mg/kg/day (2 mL mad honey dissolved in distilled water)	3 days	Male albino Wister rats	Post-treatment blood glucose level (after administration of mad honey for 3 consecutive days) was markedly reduced in both streptozotocin-induced diabetic rats and control groups.
Hemmati et al. (2015) [46]	Honey from jujube plant area, in South Khorasan, Iran	1.0 g and 2.0 g/kg body weight/day	21 days	Adult male Wistar rats	Diabetic rats supplemented with honey (1 and 2 g/kg) had significantly lower FBS (7.8 ± 0.12 mmol/L and 9.03 ± 0.15 mmol/L, respectively) than diabetic control rats (31.1 ± 2.3 mmol/L). Honey significantly increased serum adiponectin (4.5 ± 0.26 mg/L) levels in diabetic rats.
Erejuwa et al. (2010) [49]	Tualang honey	1.0 g/kg	28 days	Male Sprague-Dawley rats	Rats treated with honey had significant lower FBS (median (IQR): 8.8 (5.8) mmol/L) compared to diabetic rats given distilled water (median (IQR): 17.9 (2.6) mmol/L).
Erejuwa et al. (2016) [50]	Natural honey (supplied by Ebonyi State, Nigeria)	1.0, 2.0 or 3.0 g/kg body weight/day	21 days	Wistar rats	Honey supplementation at 1.0 and 2.0 g/kg/day significantly reduced FBS level in diabetic rats induced with alloxan (*p* < 0.05).
Sheriff et al. (2011) [51]	Unknown honey species	1.0 mL/200 g body weight	7 days	Male albino rats	Treatment with honey resulted in a non-significant reduction of FBS and 2 hour postprandial glucose level compared with untreated alloxan-induced DM rats (8.44 ± 1.66 and 11.05 ± 2.11 mmol/L vs. 11.57 ± 2.22 and 16.45 ± 3.11 mmol/L, respectively).
Arabmoazzen et al. (2015) [52]	Honey from a bee keeping center of Urmia city, Iran	3 mL/kg, 5% honey solution given 3 times/day	56 days	Adult male Wistar rats	After 56th days, serum glucose concentration of noise-induced diabetic rats treated with honey had a significant lower concentration (208 ± 34.6 mg/dL) (*p* < 0.01) compared to untreated diabetic rats. Honey-treated rats had a larger diameter of the Langerhans islands in the pancreas compared to diabetic control (5.6 ± 154.5 vs. 4.75 ± 54.25 μm)
Al-Waili (2004) [53]	Natural honey	90 g honey in 250 mL water.	Once (30 min prior to blood sampling)	7 patients of type II DM	Patients with type II DM had significantly lowered blood glucose elevation when administered with honey compared to dextrose.
Krishnasree & Mary (2017) [59]	Multifloral honey (*Apis cerana indica* F., *Apis mellifera* L., *Apis dorsata* F., *Apis florea* F. and *Trigona iridipennis* S.)	100–500 μg/mL concentration	-	In vitro assays for α-amylase and α-glucosidase inhibitory activities	*Trigona iridipennis* honey had the strongest α-amylase and α-glucosidase inhibitory properties compared to other multifloral honey species. Inhibition of α-amylase enzyme was comparable to standard diabetic therapy by acarbose especially the highest concentration of 500 μg/mL.

DM, diabetes mellitus; FBS, fasting blood sugar; IQR, interquartile range.

**Table 3:** Hypolipidemic effects of honey in clinical and pre-clinical studies.

Author	Honey Sources/Types	Dose	Duration	Models	Results
Aziz et al. (2017) [13]	Stingless bee honey (SLBH)	1.0 or 2.0 g/kg/day	28 days	Male Sprague-Dawley rats	Treatment with 2.0 g/kg of SLBH significantly reduced TG, TC and LDL levels in diabetic compared to untreated diabetic rats (*p* < 0.05). Treatment with 2.0 g/kg of SLBH significantly increased HDL levels in diabetic rats (*p* < 0.05).
Nemoseck et al. (2011) [16]	Clover honey from Hunter’s Honey Farm, Martinsville, Ind, United States	Honey diluted with water with energy density of 11.41 kJ/g	33 days	Male Sprague-Dawley rats	Rats given honey have lower TG levels (54.4 ± 19.3 mg/dL vs. 77.2 ± 36.7 mg/dL) (*p* ≤ 0.05).
Chepulis & Starkey (2008) [31]	Honeydew honey	10% honey (100 g/kg) mixed in diets	365 days	Sprague-Dawley rats	Honey fed rats had: Significantly higher HDL levels (2.82 ± 0.30 mmol/L) than sugar-free diet (2.32 ± 0.33 mmol/L) and sucrose diet (2.44 ± 0.51 mmol/L). Non-significant decreases of TG and LDL.
Yaghoobi et al. (2008) [33]	Natural unprocessed honey (unknown type)	70 g honey dissolved in 250 mL tap water daily	30 days	60 overweight or obese (BMI > 25 kg/m^2^) individuals (age: 20–60 years old)	Honey supplementation led to: Significantly lowered TG levels by 19% (*p* = 0.006) and non-significantly reduced TC by 3.3% and LDL by 4.3% in dyslipidemic subjects. Non-significantly reduced TC (3%), LDL (5.8%), TG (11%), and increased HDL (3.3%) in non-dyslipidemic individuals.
Bahrami et al. (2009) [34]	Natural unprocessed honey from Samans kandeh, Neka, Sari City, Iran	1st 14 days: 1.0 g/kg/day 2nd 14 days: 1.5 g/kg/day 3rd 14 days: 2.0 g/kg/day 4th 14 days: 2.5 g/kg/day	56 days	48 type II diabetes patients (25 patients were given honey; 23 patients as control)	Honey groups have significantly lower TC, LDL, TG (*p* = 0.000) and higher levels of HDL (*p* < 0.01) compared to baseline.
Öztaşan et al. (2005) [40]	Mad honey	50 mg/kg	3 days	Male albino Wistar rats	Mad honey administration significantly reduced TC, TG and VLDL in both control groups and experimental groups.
Erejuwa et al. (2016) [50]	Nigerian honey from bee farm in Ebonyi State, Nigeria	1.0 g/kg, 2.0 g/kg, 3.0 g/kg (dissolved in drinking water)	21 days	Wistar rats	In diabetic rats, honey significantly reduced TG & VLDL levels (*p* < 0.05). Honey dose of 2.0 g/kg significantly increased HDL levels in diabetic rats (*p* < 0.05). Honey dose of 1.0 & 2.0 g/kg significantly lowered non-HDL levels in diabetic rats (*p* < 0.05). Honey dose of 1.0 & 2.0 g/kg resulted in non-significant reduction of TC and LDL levels compared with non-diabetic control (*p* > 0.05).
Al-Waili (2004) [53]	Natural honey	Honey solution (75 g honey in 250 mL of water)	15 days	8 healthy volunteers and 5 dyslipidemic patients	Treatment with honey resulted in: Non-significant decreases of TC, LDL and TG with a non-significant increase of HDL in healthy volunteers. A significant decrease of TC with a non-significant decrease of LDL in dyslipidemic patients.
Busserolles et al. (2002) [55]	Honey from a local supplier in Ceyrat, France	65 g/100 g honey in purified diets	14 days	Male Wistar rats	Honey-fed rats had significantly lower TG levels (1.49 ± 0.12 mmol/L) than fructose-fed rats (2.03 ± 0.20 mmol/L) (*p* < 0.05).
Mushtaq et al. (2011) [69]	Natural Honey obtained from ‘The Beehive’ of Food and Fine Pastries Manufacturing Co., Ltd., Jeddah, Saudi Arabia	40 g honey dissolved in tap water daily	28 days	128 obese & 128 normal weight individuals from four different ethnic groups (P = Pathan; B = Baloch; H = Hazara; PU = Punjabi)	Honey significantly: Reduced TC in normal male of ethnic (P) and in normal male and female of ethnic (B). Reduced TC in obese male and female of ethnic (B, PU) and obese female of (H). Reduced TG in normal male (B) and normal female of (PU). Reduced TG in obese male in all ethnic groups and all obese female ethnic groups except (H). Increased HDL in normal male of (P, PU). Increased HDL in both gender of obese (B, P) and obese male of (H). Reduced LDL in obese male in all ethnic groups and obese female of (B, P).
Münstedt et al. (2009) [70]	Mixed blossom honey from Europe, Central America, and South America	75 g	14 days	60 patients with hypercholesterolemia (30 patients were given honey; 30 patients were given honey-comparable sugar solution)	Only female patient receiving honey had significantly reduced LDL compared to sugar solution group.
Khalil et al. (2015) [74]	Tualang honey	3 g/kg/day	45 days	Male albino Wistar rats	Pre-treatment with honey significantly lowered serum total cholesterol (TC) and triglyceride (TG) levels than untreated rats with ISO-induced myocardial infarction (*p* < 0.05).

BMI, body mass index; HDL, high-density lipoprotein; ISO, isoprenaline; LDL, low-density lipoprotein; SLBH, stingless bee honey; TC, total cholesterol; TG, triglyceride; VLDL, very low-density lipoprotein.

**Table 4:** Antihypertensive effects of honey in clinical and pre-clinical studies.

Author	Honey Sources/Types	Dose	Duration	Models	Results
Erejuwa et al. (2012) [14]	Tualang honey	1 g/kg	84 days	WKY and SHR	The elevated SBP in SHR was significantly reduced when treated with TH (*p* < 0.01). Honey supplementation did not affect SBP in normotensive WKY.
Erejuwa et al. (2011) [84]	Tualang honey	1 g/kg	21 days	STZ-induced diabetic WKY and SHR	TH reduced blood pressure in STZ-induced diabetic SHR, but not in STZ-induced diabetic WKY (*p* < 0.01).
Yong et al. (2016) [87]	Tualang honey	0.01% to 1%	Human umbilical vein endothelial cells (HUVECs) were pre-treated with TH for 4 h	HUVECs	TH suppressed the actin remodelling, caveolin-1 production and reduced the disruption of endothelial adherence junctions thus inhibiting H_2_O_2_-induced endothelial permeability
Romero-Silva et al. (2011) [91]	Unknown honey species	20% added in diet	56 days	Wistar rats	Rats given honey in their hypercaloric diet showed no increase in blood pressure compared to rats that did not receive honey (*p* < 0.05).
Al-Waili (2003) [92]	Unknown honey species	Inhalation of honey solution (60% *w*/*v*)	Once for 10 min	24 healthy individuals, 16 type II DM patients and 6 hypertensive patients	Blood pressure was decreased in hypertensive patients significantly at 60 and 120 min after honey inhalation.
Aluko et al. (2013) [93]	Faculty of Agriculture of the University of Ilorin, Nigeria	20 mL	Once	100 individuals (50 males and 50 females)	Honey administration significantly reduced SBP in males, and females.
Aluko et al. (2014) [94]	Faculty of Agriculture of the University of Ilorin, Nigeria	20 mL	Once	50 males	Honey significantly decreased SBP after 15, 30 and 60 min of consumption compared to baseline value (110.20 ± 2.14 mmHg, 111.33 ± 2.14 mmHg, 110.4 ± 2.08 mmHg vs. 117.80 ± 0.88 mmHg)

DM, diabetes mellitus; H_2_O_2_, hydrogen peroxide; HUVEC, human umbilical vein endothelial cells; SBP, systolic blood pressure; SHR, spontaneously hypertensive rats; STZ, streptozotocin; TH, tualang honey; WKY, Wistar-Kyoto rats.
